# MARITAL STATUS DIFFERENCES IN SOCIAL ISOLATION IN LATER LIFE

**DOI:** 10.1093/geroni/igae098.0104

**Published:** 2024-12-31

**Authors:** Lydia Li, Sara McLaughlin

**Affiliations:** University of Michigan, Ann Arbor, Michigan, United States; Miami University, Oxford, Ohio, United States

## Abstract

Social isolation, characterized by infrequent social contact, is associated with morbidity, mortality, and healthcare costs. Using 9 waves of data from the National Health and Aging Trends Study (NHATS) that has conducted annual interviews with a probability sample of Medicare beneficiaries since 2011, this study examines how social isolation differs by marital status and whether the association between marital status and social isolation varies by gender. The sample includes 7,077 respondents aged 65+. We examine 4 categories of marital status (coupled, widowed, divorced, and never married.) Social isolation is assessed by an established index that uses four domains (living arrangements, close networks, religious service attendance, and participation in other activities) to determine social isolation (yes and no). Covariates include age, gender, race, education, community/facility residence, and time. Multilevel logistic regression is conducted. Results show that the risk of social isolation, from low to high, is coupled, widowed, divorced, and never married. The difference between all pairs, except divorced and unmarried, is statistically significant. The interaction effects of marital status and gender on social isolation are statistically significant [F (3, 54) = 3.43, p=.02]. Specifically, the probability of social isolation for coupled, widowed, divorced, and never married is.19,.35,.49, and.52 in men and.09,.25,.29, and.32 in women. The findings imply that divorced and never-married men should be prioritized for social isolation reduction interventions.

